# Unveiling the obesogenic neighborhood food environment factors and typologies in Tianjin, China: an integrative analysis of perceived and objective measures

**DOI:** 10.3389/fpubh.2025.1665021

**Published:** 2025-11-21

**Authors:** Yue Sun, Wei Lu, Jinyuan Gu, Yishu Yao, Tianyue Wan

**Affiliations:** 1 Research Section of Environment Design, School of Architecture and Fine Art, Dalian University of Technology, Dalian, China; 2 College of Landscape Architecture and Art, Fujian Agriculture and Forestry University, Fuzhou, China

**Keywords:** neighborhood food environment, overweight and obesity, perceived measurement, objective assessment, food deserts

## Abstract

**Introduction:**

Assessing and intervening in food environments constitutes a critical strategy for addressing the obesity epidemics. However, existing assessments predominantly focus on either objective or perceived dimensions, with limited attention to developing countries. This study investigates the impact of neighborhood-level food environments on resident obesity in a national central city of China and establishes a typology of obesogenic community profiles.

**Methods:**

We developed an integrative tool that harmonizes objective geospatial data with subjective perceptual metrics. Leveraging stratified sampling survey data on neighborhood food environments (*N* = 405) and multiscale geospatial datasets from Tianjin, China (2023), we establish a comprehensive indicator repository for neighborhood food environments. Dimensionality reduction via principal component analysis (PCA) was applied to all measured indicators, followed by an ordinal multinomial regression model to identify significant obesogenic determinants at the neighborhood level. Finally, the K-means clustering algorithm was subsequently implemented to delineate prototypical obesogenic neighborhood typologies.

**Results:**

Among 10 principal components derived from PCA, four obesogenic factors were identified, ranked by effect magnitude: FAC_8 (Perceived Community Food Accessibility Index, *β* = −0.382, *p* = 0.001, OR = 0.68), FAC_4 (Food Availability and Diversity within 500-1000m, *β* = 0.225, *p* = 0.061, OR = 1.25), FAC_6 (Unhealthy Dietary Behavior, *β* = −0.191, *p* = 0.066, OR = 0.68), and FAC_3 (Retail Food Environment Index within 500m, β = −0.184, *p* = 0.078, OR = 0.83). K-means clustering delineated three obesogenic neighborhood types: Objective Deprived (N = 10, 6.1%), Objective Overloaded (*N* = 37, 22.56%), and Objective Overloaded-Dietary Behavior Integrated (*N* = 117, 71.34%).

**Discussion:**

This study revealed that within the context of China’s urban built environment, the prevalence of “food deserts” is minimal. Conversely, an augmented proportion of widely recognized healthy food facilities in developed Western countries has been observed to heighten the risk of obesity, including supermarkets and fresh food markets. This phenomenon exhibits a scale-dependence, indicating that its impact increases with the magnitude of the scale. The most salient characteristic of obesogenic neighborhoods in China is their high objective environmental risk. The study examined and identified neighborhood-level obesity factors and provided a generalizable method for identifying obesogenic neighborhood types, thereby providing empirical evidence for obesity research in developing countries.

## 1 Introduction

Globally, obesity and its associated metabolic syndromes and cardiovascular diseases are proliferating at an alarming rate as critical public health crises. World Health Organization data indicates a near-tripling of global obesity prevalence since 1975, with urban populations constituting over 75% of cases. The etiology of obesity spans multidimensional determinants ranging from individual stress-related eating behaviors (1), and sociocultural evolution (2) to built environment characteristics (3), Environmental modification strategies demonstrate greater feasibility and efficacy compared to individual-centric lifestyle interventions (4). The retail food environment, as a critical built environment component, exhibits strong associations with dietary patterns: Limited access to healthy foods may precipitate adverse dietary behaviors and health outcomes (5, 6). Consequently, community-level food environment optimization has been widely advocated as a strategic obesity intervention (7).

Low-quality food environments are typically categorized as “food deserts” (8) or “food swamps” (9). The former concept, originating from a 1990 Scottish government publication, describes areas with limited access to healthy foods, frequently operationalized through metrics assessing retail food availability (e.g., supermarket scarcity versus convenience store predominance) (10). The latter term, emerging from 2009 U. S. scholarship, characterizes environments where energy-dense food options overwhelm healthy alternatives, exacerbating nutritional risks (11). Concurrently, the “obesogenic environment” framework proposed by Swinburn et al. (12) through the ANGELO (Analysis Grid for Environments Linked to Obesity) model provides a holistic conceptualization of environmental obesogenicity. However, these constructs predominantly reflect developed nations’ contexts, and their applicability to, for example, non-developed Asian countries is difficult to ascertain (13). While significant spatial disparities persist across urban–rural gradients (14), national boundaries (15), and income strata (16). Notably, low- and middle-income countries (LMICs) face escalating obesity burdens yet remain critically understudied (constituting merely 10% of research output) (17), with scant evidence linking food environment exposures to health outcomes (16).

In China, obesity has emerged as a paramount public health challenge amidst the dual burden of undernutrition and overnutrition (18, 19). Distinct from Western contexts, Chinese urban food environments and dietary cultures demonstrate unique sociocultural configurations (20), While consensus exists regarding food environment-obesity associations, critical knowledge gaps persist: (1) Limited evidence on specific food environment typologies’ differential impacts (21); (2) Methodological bifurcation between perceived versus objective measurement approaches (22–25); and (3) Absence of integrated assessment frameworks.

Objective measurement dominates food environment research (>60% of studies) through GIS-based analyses and statistical indicators (e.g., food outlet density within buffer zones) (26–28), while enabling standardized spatial quantification, this approach neglects individual-level behavioral mediators—for instance, temporal or economic constraints altering accessibility perceptions despite equivalent spatial proximity (29, 30). Perceptual assessments, though underutilized, capture subjective experiences and preferences, offering complementary insights (31, 32). The synergistic integration of both paradigms remains empirically underexplored.

Building upon this foundation, our study investigates the obesogenic food environment in Tianjin—a Chinese metropolis with distinct dietary patterns—through a dual-measurement integrative lens. In particular, it is emphasized that in our study, “food environment” is operationally defined as neighborhood-level points of interest (POIs) associated with food retail facilities, while regionally embedded culinary cultural landscapes are explicitly excluded from the scope of investigation. We address three core inquiries as follows: (1) Do objective food environments, perceived food environments, and dietary behaviors all influence weight outcomes among Chinese? (2) Based on the positive findings from the first question, *what are the key environmental factors and individual-level determinants influencing weight outcomes?* (3) Based on the evidence from Question 1 and Question 2, how can we classify and assess the overall obesity risk status of typical Chinese urban communities?

## 2 Study area and methods

### 2.1 Study area and participants

**Figure 1:**
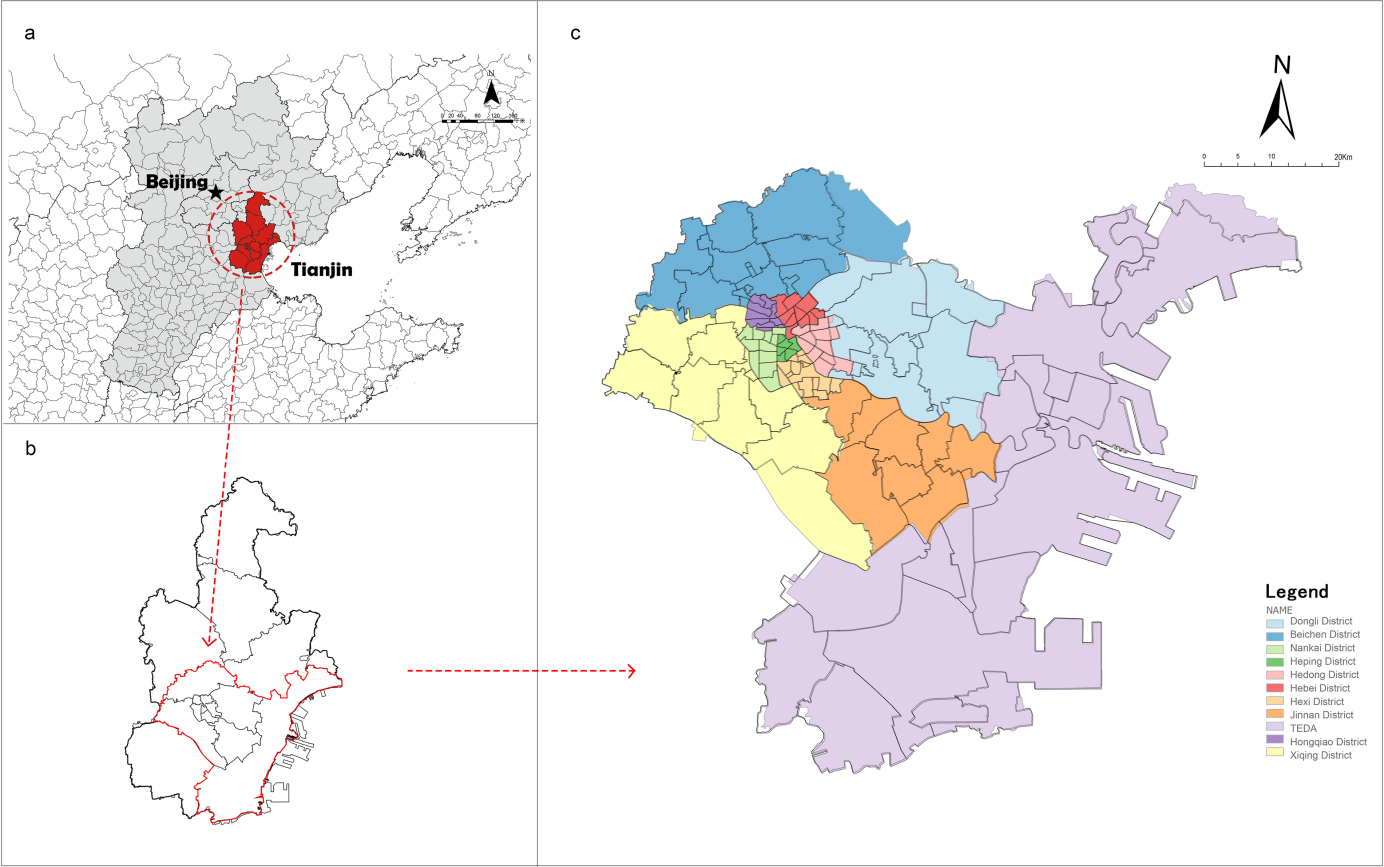
Study area. **(a)** Circum-Bohai sea economic zone, China. **(b)** Tianjin, Circum-Bohai sea economic zone, China. **(c)** Main urban, suburban and TEDA (Tianjin economic-technological development area), Tianjin.

This study utilized a cross-sectional survey conducted from January to March 2023 in the main urban area of Tianjin, China (Figure 1). The research focused on the metropolitan core of Tianjin (38°34′–40°15′N, 116°43′–118°04′E), a critical coastal hub connecting the Beijing-Tianjin-Hebei urban agglomeration and Northeast Asia. Characterized by its 153-kilometer Bohai Sea coastline and Haihe River Basin, Tianjin exhibits a unique dietary culture shaped by its geographical advantages and historical urban development, featuring abundant riverine and marine delicacies, as well as poultry and game meats (33). Notably, Tianjin ranks third in China for overweight and obesity prevalence (64), making it an ideal case for investigating dietary-environment interactions. The study area encompassed central urban districts and four suburban zones (including Binhai New Area), yielding 405 valid questionnaires. This region represents 72.22% of Tianjin’s population, ensuring demographic representativeness and high participant engagement.

The study adopts residents’ daily living circles as the analytical scale, operationalized through ArcGIS-based buffer analysis. Specifically, we generated buffer maps in ArcGIS with 300m, 500m (primary), and 1000m radii to simulate 5-, 10-, and 15-min urban pedestrian catchments—thresholds aligned with China’s official daily living circles standards for neighborhood service accessibility (35). Preliminary analyses revealed limited food environment exposure within 300m buffers, attributable to the prevalence of large-scale gated communities in Chinese cities that create spatial discontinuities in facility distribution. Consequently, the 300m scale was excluded from subsequent analyses. Final operationalization employed 500m and 1000m buffers to represent: High-frequency pedestrian food procurement zones (daily walking accessibility) and Periodic procurement corridors (walking/short-drive accessibility). The prioritization of pedestrian metrics reflects empirical evidence that walking constitutes the predominant mode for food acquisition in Chinese urban contexts (36). This multiscalar approach captures the hierarchical structure of food environment exposure while addressing the morphological specificities of Chinese urban form.

### 2.2 Data measurement

Objective food environment data were derived from open-source geospatial databases and survey questionnaires. Community-level spatial data, including retail and food service facilities, were obtained from OpenStreetMap, the Chinese Academy of Sciences Data Center,^1^ and the Tianjin Municipal Open Data Platform. After data cleaning—which removed 891 irrelevant entries—75,079 valid points of interest (POIs) were retained, comprising 15,959 food retail facilities and 59,120 food service establishments (Table 1).

**Table 1:** List of POIs of food facilities within the study area.

Level I	Level II	Level III	Quantity (bars)	Total (bars)
Food retail facilities	Supermarkets	X1 supermarket	68	15959
	Vegetable market/farmer’s market	X2 vegetable market/farmer’s market	5034	
	Community supermarkets/grocery stores	X3 community supermarket/grocery store	2724	
	Convenience Store	X4 convenience store	2981	
	Fresh food shops	X5 fresh food speciality store	5152	
Catering facilities	Takeaway restaurants	X6 Takeaway Restaurants	8086	59120
	Fast food restaurants	X7 fast food—Chinese	8464	
		X8 fast food—western	325	
	Restaurant Ching	X9 restaurant-Chinese	34043	
		X10 restaurant-western	2544	
	Dessert shop	X11 dessert shop	5658	

Perceived community food environment data originated from the 2023 Tianjin Health Survey (*N* = 405 valid responses; 55.0% online and 81.9% offline response rates; overall 73% validity deemed acceptable), a structured questionnaire comprising four modules: Individual socioeconomic attributes, Community food environment perceptions, dietary behavior patterns, and health status indicators (the questionnaire can be found in Supplementary Material 1). Data collection employed a hybrid web-based (via Credamo, a questionnaire data platform from China, the link is: https://www.credamo.com/#/) and face-to-face (street-intercept sampling) protocol. All participants provided written informed consent prior to engagement and received ¥5 RMB monetary compensation. Final analytical samples included 405 validated responses, with differential validity rates across modalities: Online surveys: 55.0% validity (platform-mediated recruitment), Offline surveys: 81.9% validity (controlled field sampling). The validity discrepancy primarily stemmed from performance on an embedded attention-check question, where offline participants demonstrated superior engagement. The aggregate validity rate of 73% meets methodological acceptability thresholds for community-level observational studies.

#### 2.2.1 Outcome variable

The study operationalized body weight outcomes at the individual level as the primary dependent variable. Specific measurements included self-reported height, weight. Body Mass Index (BMI) was calculated to two decimal places using the Equation 1:

$$\mathrm{BMI}=\frac{\text{Weight}(\mathrm{kg})}{\text{height}{\left(m\right)}^{2}}$$ (1)

Classification followed the Chinese BMI classification, BMI < 18.5 denotes underweight, 18.5 ≤ BMI < 24.0 denotes normal weight, 24.0 ≤ BMI < 28.0 denotes overweight, and BMI ≥ 28.0 denotes obesity. The WHO standard defines adult overweight as BMI 25.0–29.9 kg/m^2^, and obesity as BMI ≥ 30.0 kg/m^2^. However, accumulated evidence indicated that variations in the association between BMI and health risks, such as body composition (e.g., body fat percentage, muscle mass), across different ethnicities and populations (37). Standards in this research developed based on epidemiological data specific to the Chinese population can more accurately identify health risks associated with overweight and obesity in this demographic. To intuitively reflect the graded health risks associated with each BMI category, we also present the assessment scoring system recommended by the Chinese Guidelines for the Prevention and Control of Overweight and Obesity in Adults (38). This system assigns a score of 100 for normal weight (G1 = 100), 80 for overweight (G2 = 80), and 60 for obesity (G3 = 60), which quantitatively signifies a decline in health status across categories. For the purpose of all subsequent regression analyses, BMI was treated as a categorical variable. The scoring is presented here for descriptive clarity and to align with the public health practice in the study context; it was not used as a continuous variable in statistical models.

#### 2.2.2 Explanatory variable

##### 2.2.2.1 Objective community food environment metrics

The accessibility of objective community food facilities was measured by calculating the point density of facility categories within three buffer zones. Equation 2 is defined as:

$$\rho_{p}(r)=\frac{N_{p}(r)}{\pi r^{2}}$$ (2)

where $N_{p}(r)$ represents the number of food facility POIs within a circular buffer of radius r centered at coordinates P(X,Y) . Buffer areas $\pi r^{2}$ were derived using ArcGIS, incorporating road network accessibility and sample point locations to ensure spatial accuracy.

This study employs the modified RFEI to measure the healthiness of the objective food environment. The Retail Food Environment Index (RFEI) was originally proposed by the California Center for Public Health Advocacy and developed for the United States and Canada (39). It is defined as the ratio of less healthy food retailers (e.g., fast-food outlets and convenience stores) to healthy food retailers (e.g., grocery stores and supermarkets) within a given area (e.g., a census tract). Compared to traditional quantitative measures, the RFEI more effectively reflects the healthiness of a community’s food environment. However, to better align with the characteristics of China’s food environment, we have modified the original RFEI based on the research approach of Amin et al. (40), who proposed a machine learning-enhanced modified Retail Food Environment Index (mRFEI), to ensure its applicability in China. Equations 3–6 are as follows:

$$\text{mRFEI}=\frac{N(\mathrm{hf})}{N(\mathrm{hf})+N(\;\mathrm{uf})}$$ (3)

$$\text{mRFEI}\_\text{Groceries}=\frac{N(\mathrm{hf})+N(\text{Groceries})}{N(\mathrm{hf})+N(\;\mathrm{uf})}$$ (4)

$$\text{mRFEI}\_\text{Convenience Store}=\frac{N(\mathrm{hf})+N(\text{Convenience Store})}{N(\mathrm{hf})+N(\;\mathrm{uf})}$$ (5)

$$\text{mRFEI}\_\text{Chinese Restaurant}=\frac{N(\mathrm{hf})+N(\text{Chinese Restaurants})}{N(\mathrm{hf})+N(\;\mathrm{uf})}$$ (6)

Where N(hf) represents the number of healthy food retailers, including supermarkets, wet markets and fresh food specialty stores. N(uf) represents the number of unhealthy food retailers, including the other food retailers. N(Groceries) represents the number of Groceries. N(Convenience Stores) represents the number convenience stores. N(Chinese Restaurants) represents the number of Chinese restaurants.

The diversity index is a measure of differences in the number and type of different food facilities in a community food environment. In landscape ecology, diversity indices are measured in a variety of ways, among which, Diversity was quantified using the Shannon-Wiener Index, adapted from ecological studies (41). The Shannon-Weiner Index was used to calculate the diversity of food facilities at different ranges of measurement for the food facility diversity studied in this research, and Equation 7 is as follows:

E=−∑[(pi)×In(pi)] (7)

Where E represents the diversity index, and pi denotes the proportion of the I-th facility type relative to the total facilities *N* in the community (e.g., pi=ni/N ).

##### 2.2.2.2 Perceived neighborhood food environment metrics

Perceived food environment data were collected via the 2023 Tianjin Community Food Environment Survey, employing a five-dimensional framework (42), which includes perceived availability, perceived accessibility, perceived affordability, perceived adaptability and perceived serviceability. Responses were recorded using 7-point Likert scales (1 = strongly disagree; 7 = strongly agree). Detailed metrics and survey items are outlined in Table 2.

**Table 2:** Measurement indicators and enquiry methods of the five-dimensional perception questionnaire.

Type	Target layer	Indicator description	Evaluation criteria
Residents’ subjective perception of the neighborhood food environment	Perceived availability	F1-Nutritious food can be easily purchased in this neighborhood, e.g., a full range of foods such as staple foods, main dishes and side dishes.	Using a 7-point Richter scale: Strongly disagree = 1, Strongly agree = 7
	Perceived accessibility	F2-It is easy to buy daily food on foot, and there is good transport to get to the food facilities, so there are no inconveniences in daily shopping	
	Perceived affordability	F3-Nutritionally balanced food is available in the neighborhood at more affordable prices	
	Perceived accommodation	F41-The opening hours and service of facilities such as supermarkets or grocery shops are satisfactory when buying necessary ingredients/food in the neighborhood	
		F42-The environmental quality of facilities such as supermarkets or grocery shops is satisfactory when buying necessary ingredients/food in this neighborhood	
		F4- Satisfactory service quality of facilities such as supermarkets or grocery shops when buying necessary ingredients/food in this neighborhood	
	Perceived acceptability	F51-The quality and appearance of ingredients/food bought in this neighborhood are satisfactory.	
		F52-I feel confident that there are trustworthy merchants and producers in this neighborhood in terms of food safety	
	Perceived availability	R1-There are a lot of restaurants around where I live that are easy to find that offer a full range of foods that are nutritious, e.g., starters, mains and side dishes;	
	Perceived accessibility	R2-It is easy to walk to nearby restaurants and there is good transport to restaurants, so there are no inconveniences to daily meals R3-I can buy well-balanced food at a relatively affordable price at nearby restaurants	
	Perceived affordability	R3-I can buy well-balanced food at a reasonable price at nearby restaurants.	
	Perceived accommodation	R41-Satisfactory opening hours and service when I want to eat in a restaurant.	
		R42-The quality of the environment in the restaurant is satisfactory when I want to eat in the restaurant	
		R43-The level of service quality in the restaurant is satisfactory when I dine in the restaurant	
	Perceived acceptability	R51-The quality and taste of ingredients/dishes served in restaurants in this neighborhood is satisfactory	
		R52-Feeling confident about the food safety in the neighborhood, with more established businesses and producers that I can trust.	

##### 2.2.2.3 Dietary behavior metrics

Dietary behavior serves as a critical determinant of body weight outcomes and has been empirically validated as a mediator between built environments and individual health outcomes (43). Drawing from established domestic measurement frameworks, this study operationalized dietary behavior through four dimensions: *dietary diversity*, *food procurement patterns*, *dining locations*, and *dietary content*. A one-week dietary recall method was employed, with participants reporting:

- Dietary diversity: Assessed via a 7-point Likert scale (1 = “extremely monotonous” to 7 = “extremely diverse”) based on self-evaluations of meal variety over the preceding week.
- Food procurement patterns: Habitual channels for food acquisition (e.g., markets, online platforms).
- Dining locations: Frequency of meals consumed at home, workplaces, or commercial establishments.
- Dietary content: Self-reported frequency and portion sizes of both healthy (e.g., vegetables, whole grains) and unhealthy foods (e.g., sugary beverages, processed snacks) consumed during the recall period.

#### 2.2.3 Control variables

In addition to the above variables, this study included individual demographic information of the participants who took part in the questionnaire, which was divided into personal information and household information, including gender, age, household registration, levels of education and chronic disease history. The household information includes the geographical location of the neighborhood, size of the household, annual household income, whether they own a private car, and employment status.

### 2.3 Methodology

#### 2.3.1 Study design

**Figure 2:**
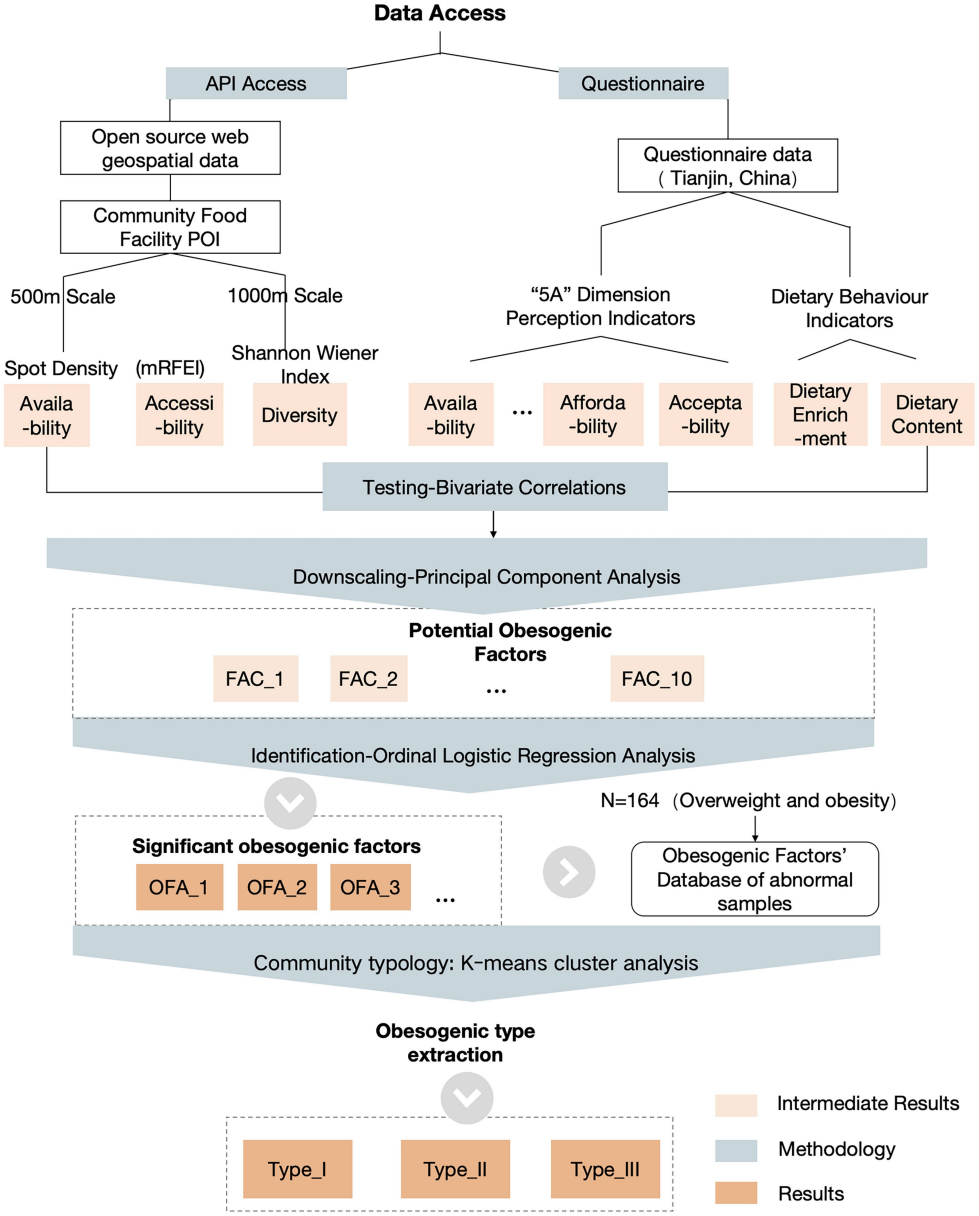
Study design framework and technical path.

Guided by socioecological theory (63), this study investigates how socioecological frameworks influence individual health outcomes through four core propositions: (1) environmental impacts on health behaviors are multifaceted and operate across multiple levels; (2) factors at different levels and dimensions interact dynamically; (3) hierarchical distinctions exist among systems, necessitating multi-level environmental interventions to effectively modify health beliefs and behaviors. The analytical framework focuses on the effects of objective food environments and perceived food environmental factors on obesity outcomes, with measurement indicators illustrated in Figure 2.

#### 2.3.2 Data analysis

This section delineates the sequential analytical strategy employed to address the study’s core research questions. Initially, bivariate correlation analysis was employed to assess individual associations and ordinal weighting outcomes among each initial variable across three conceptual dimensions (objective, perceived, and behavioral). Subsequently, principal component analysis (PCA) was conducted to mitigate multicollinearity and identify key latent factors for robust modeling. A simplified set of orthogonal factors derived from PCA was then utilized in multivariate ordinal logistic regression models. Controlling for sociodemographic covariates, the simultaneous influence of the three dimensions on weight outcomes was tested, addressing Question 1. The output from the ordered logistic regression model identified key obesity-promoting factors, directly addressing Question 2. Finally, K-means clustering was employed for standardization and integration, enabling community classification by assessing shared obesity risk profiles.

##### 2.3.2.1 Bivariate correlation

Bivariate correlation analysis was conducted to measure the strength and direction of the relationships between the objective food environment, perceived food environment, individual dietary behaviors, and weight outcomes (addressing Research Question 1). We employed SPSS 28.0 software to conduct bivariate analyses on all initial variables. This served two purposes: firstly, to examine whether the objective food environment, perceived food environment, and individual dietary behaviors were associated with weight outcomes; secondly, to preliminarily assess whether multiple factors presented a risk of multicollinearity.

##### 2.3.2.2 Multifactor dimensionality reduction

After analyzing the multicollinearity test of the 38 measures obtained from the above pathway measurements, we found that these measures have multicollinearity problems within the three dimensions (objective indicator pool, perception indicator pool, and eating behavior indicator pool). Principal Component Analysis (PCA) is a commonly used dimensionality reduction method on medium-sized datasets and non-sparse dataset scenarios, transforming high-dimensional data into low-dimensional data through linear transformation. The study utilizes SPSS 28.0 software for Principal Component Analysis (PCA) of indicators to organize and merge these potential indicators to improve the data processing efficiency of the subsequent study.

##### 2.3.2.3 Identification of obesogenic factors

Given the ordered categorical nature of the dependent variable (weight outcomes: G1 = 100, G2 = 80, G3 = 60), ordinal logistic regression was employed to identify obesogenic factors while controlling for sociodemographic covariates. This method is preferred over linear regression for ordinal outcomes, as it models cumulative probabilities across response categories. The logit function is expressed as:

For each ordered category j=1,2,⋯,K−1 of the dependent variable Y, the model establishes a relationship through a Logit link function of cumulative probabilities (Equation 8):

$$\text{logit}(P(Y\leq j|X))=a_{j}+\beta_{1}X_{1}+\beta_{2}X_{2}+\cdots +\beta_{p}X_{p}$$ (8)

where Y is an ordered categorical dependent variable (body weight outcomes) with values of 1, 2. K. $a_{j}$ is the intercept term for category j (needs to satisfy $a_{1}<a_{2}<\cdots <a_{k-1}$ ). where $X_{1},X_{2}\cdots X_{p}$ represents the independent variables (objective food environment factor, perceived food environment factor, and eating behavior factor). $\beta_{1},\beta_{2}\cdots \beta_{p}$ are regression coefficients for the independent variables.

##### 2.3.2.4 Probability calculation

The probability of each category is derived from the difference in the cumulative probabilities (Equation 9):

$$(Y=j\;|\;X)=\{\begin{array}{c} P(Y\leq j\;|\;X)-P(Y\leq j-1|\;X)\;\;\;\text{When}\;1<j<K\\ P(Y\leq 1\;|\;X)\;\text{When}\;j=1,\\ 1-P(Y\leq K-1|\;X)\;\;\text{When}\;\;j=K. \end{array}$$ (9)

where the accumulation probability P(Y≤j∣X) is computed via an inverse logit function (Equation 10):


$$P(Y\leq j\;|X)=\frac{1}{1+\exp(-(a_{j}+\beta_{1}X_{1}+\cdots +\beta_{p}X_{p}))}$$ (10)

##### 2.3.2.5 Neighborhood typology and evaluation

Existing studies classify obesogenic environments primarily at the facility level, neglecting systemic neighborhood-scale assessments. To address this gap, we developed a neighborhood obesogenic risk evaluation model via the following workflow:

*Data standardization:* We extracted 164 obesogenic factors from the sample of weight outlier samples [overweight (*N* = 131, 79.8%), obese (*N* = 33, 20.2%)] for classification. Since the scale and positive and negative orientation of each indicator in the indicator system are different, it is necessary to standardize the indicators before downgrading the obesogenic factors for subsequent comprehensive evaluation. The specific processing Equations 11–12 is as follows:


$$\mathrm{Positive indicators:}X_{\mathrm{ij}}=\frac{X_{\mathrm{ij}-\min(X_{\mathrm{ij}})}}{\max(X_{\mathrm{ij}})-\min(X_{\mathrm{ij}})}$$ (11)

$$\mathrm{Negative indicators:}X_{\mathrm{ij}}=\frac{\max(X_{\mathrm{ij}})-X_{\mathrm{ij}}}{\max(X_{\mathrm{IJ}})-\min(X_{\mathrm{ij}})}$$ (12)

where $X_{\mathrm{ij}}$ denotes the standardized value of the j-th indicator in the i-th dimension. The data were processed in SPSS 28.0 to generate a harmonized dataset.

*K-means clustering:* K-means clustering was first determined by contour coefficients together with the elbow rule, using the SSE value of each cluster calculated from 2–28; according to the elbow rule, the inflection point of the SSE value was selected as the optimal number of clusters. Then, K-means clustering analysis was carried out in the following steps: select the initialized k samples as the initial clustering centers $a=a_{1},a_{2}\cdots a_{k}$ for each sample in the dataset, calculate its distance to the k clustering centers and classify it into the class corresponding to the clustering center with the smallest distance; for each category $a_{j}$ , recalculate its clustering centers (Equation 13):

$$a_{j}=\frac{1}{|c_{i}|}\sum_{x\in c_{i}}x$$ (13)

Then, steps 2–3 are repeated until convergence is reached (max number of iterations = 100; tolerance = 10^5^).

## 3 Results

### 3.1 Descriptive statistics

Table 3 presents the postcleaning descriptive statistics for all the variables, including the means, standard deviations, skewness, and kurtosis. The absolute skewness values ranged from 0.024 to 1.80, and the kurtosis values ranged from 0.06 to 4.56, all within acceptable thresholds (skewness < ±2, kurtosis < ±7), indicating no significant deviation from normality in the data distribution.

**Table 3:** Descriptive statistics of the socioeconomic attributes of the sample.

Variable	Mean value	Average	Median	Standard	Skewness	Kurtosis
Gender	Male = 1, Female = 0	0.45	-	0.50	0.184	−1.976
Age^1^	Age of participants	-	3	1.30	0.346	−0.967
Household registration	Local domicile = 1, other domicile = 0	0.79	-	0.41	−1.449	0.099
Levels of education^2^	Levels of education to the highest level of qualification	-	16	2.49	0.957	1.231
Household size	Number of family members living together	3.15	-	0.99	0.480	0.945
Annual household income^3^	Participants’ total annual household income	-	3	1.31	0.130	−0.979
Own a private car	Own a private car = 1, no private car = 0	0.74	-	0.44	−1.103	−0.788
Employment status	Be on board = 1, other = 0	0.87	-	0.33	−1.263	2.159

^1^This variable is an ordinal categorical variable, under 18 = 1, 18−29 = 2, 30–39 = 3, 40–49 = 4, 50–59 = 5, 60 and above = 6. 50 ~ 59 years old = 5, 60 years old and above = 6. ^2^This variable is an ordinal categorical variable, primary school and below = 1, junior secondary school = 2, senior secondary school/vocational secondary school = 3, college/higher vocational colleges = 4, Bachelor’s degree = 5, postgraduate (master’s degree and above) = 6. ^3^This variable is an ordinal categorical variable, less than 30,000 yen = 1, 30–99,000 yen = 2, 100–149,000 yen = 3, 15–19,000 yen = 4, 20–39,000 yen = 5, 400,000 yen and above = 6. “-” indicates meaninglessness.

The sample comprised 45% male (*N* = 184/405) and 55% female (*N* = 221/405) participants, with a median age range is 30–39 years old. Among the respondents, 79% held Tianjin *hukou* (household registration, *N* = 321/405), and (equivalent to college-level education). These sociodemographic characteristics align with Tianjin’s 7th National Population Census (34), confirming sample representativeness.

**Figure 3:**
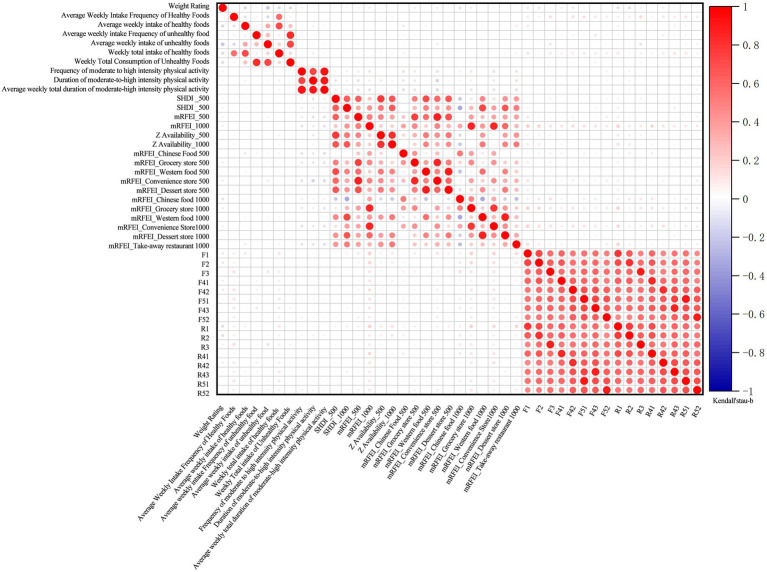
Correlation matrix of individual, behavioral, and environmental factors with weight outcomes.

Bivariate tests (Figure 3) revealed significant correlations among three core dimensions: objective food environments, perceived food environments, and dietary behaviors. These findings indicate multicollinearity among initial evaluation metrics, necessitating dimensionality reduction prior to modeling. Furthermore, these findings provide preliminary answers to question 1. We observed that perceived food environment, dietary behaviors, and physical activity all exhibit direct associations with weight outcomes. Within perceived food environments, R1-perceived availability (*β* = −0.126*) and R2-perceived accessibility (β = −0.122*) exhibited negative correlations with weight outcomes. Regarding dietary behaviors, average weekly intake of healthy food (β = −0.148*), total weekly intake of healthy food (β = −0.139**), average weekly intake of unhealthy food (β = −0.207**), and total weekly intake of unhealthy food (β = −0.138**) were negatively correlated with weight outcomes. Conversely, dietary intake diversity (β = 0.141**) showed a positive correlation with weight outcomes. Furthermore, we observed that the direct association between the objective food environment and weight outcomes was weak. However, it was associated with the subjective perception of the food environment and physical activity levels (correlation coefficients detailed in Supplementary Material 2). This suggests that the objective food environment may not directly influence weight outcomes but could exert an effect through mediating mechanisms.

### 3.2 Identification of obesogenic factors

#### 3.2.1 Dimensionality reduction

Collinearity diagnostics confirmed multicollinearity across objective/perceived food environments and dietary behavior indicators. Principal component analysis (PCA) was conducted after verifying suitability via the KMO measure: 0.776 (>0.5 threshold) and Bartlett’s Sphericity test: χ^2^ = 17,896.391, *p* < 0.001 (Table 4). The PCA extracted 10 principal components (eigenvalues >1) from 38 standardized indicators, cumulatively explaining 77.15% of the variance (Table 5). All communalities exceeded 0.5, confirming robust factor retention.

**Table 4:** KMO and Bartlett’s sphericity test results.

KMO number of sample suitability measures		0.776
Bartlett’s test of Sphericity	Approximate chi-square (math.)	17896.391
	degree of freedom (df)	903
	Significance	0

**Table 5:** Implications of principal component factors for dimensionality reduction.

Number	Factor meaning	Cumulative value in percent	Indicators included in factors
FAC_1	Integrated perceived neighborhood food environment	25.37	F3, R3, F41, R41, F42, R42, F43, R43, F5 1, R51, F52, R52.
FAC_2	1000-meter modified retail food environment index	38.99	mRFEI_1000, mRFEI_ Convenience Store 1000, mRFEI_Groceries1000.
FAC_3	500-meter modified retail food environment index	46.63	mRFEI_500, mRFEI_ Convenience Store 500, mRFEI_Groceries_500.
FAC_4	500 m-1000 m food availability and diversity	52.59	Z Availaibility_1000, Z Availability _500, SHDI_500.
FAC_5	Moderate to high intensity physical activity	58.05	Average weekly total duration of moderate-high intensity (min), frequency of moderate-high intensity physical activity (times/week), duration of moderate-high intensity physical activity (min).
FAC_6	Unhealthy eating behavior	63.32	Total weekly intake of unhealthy food, Average weekly frequency of unhealthy food intake, average weekly intake of unhealthy food.
FAC_7	Index of food facilities in Chinese restaurants in the 500 m-1000 m buffer zone	67.30	mRFEI_ Chinese Restaurants 1000, mRFEI_ Chinese Restaurants 500, SHDI_1000.
FAC_8	Perceived food availability and accessibility	70.96	F2, R2, F1, R1
FAC_9	Healthy food intake	74.32	Average weekly intake of healthy food, Total weekly intake of healthy food
FAC_10	Frequency and abundance of healthy food intake	77.15	Average weekly frequency of healthy food intake, dietary intake richness

#### 3.2.2 Analysis of obesogenic factors

An ordered logistic regression analysis was conducted between the 10 clustered factors and weight health ratings. The model demonstrated good fit (Table 6), with a significant *p* value of 0.000 (<0.05). The parallel lines assumption was tested using a Chi-Squared test. For this specific test, a non-significant result (*p* > 0.05) is desirable, as it indicates that the null hypothesis—that the slope coefficients are equal across all categories of the ordinal outcome—cannot be rejected. Thus, the result (Table 7: χ^2^ = 43.201, *p* = 0.056) supports the validity of the proportional odds assumption for the ordered logistic regression model.

**Table 6:** Model fit information.

Model	−2 Log-likelihood	Cardinality	Degree of freedom	Significance
intercept only	737.131			
final	655.727	81.403	25	0.000

**Table 7:** Parallel line test results.

Model	-2 Log-likelihood	Cardinality	Degree of freedom	Significance
Original hypothesis	646.939			
conventional	603.738b	43.201c	30	0.056

Table 8 presents the full-sample regression outcomes. The analysis identified four factors with statistically significant effects on weight outcomes. At the environmental level, two significant factors were identified: FAC_3 (500-meter modified retail food environment index: *β* = −0.184, SE = 0.104, *p* < 0.1, OR = 0.83) and FAC_4 (500–1000 m food availability and diversity: β = 0.225, SE = 0.12, *p* < 0.1, OR = 1.25). At the individual level, two significant factors emerged: FAC_6 (unhealthy eating behavior: β = −0.191, SE = 0.104, *p* < 0.1, OR = 0.83), and FAC_8 (perceived food availability and accessibility: β = −0.382, SE = 0.113, *p* < 0.001, OR = 0.68).

**Table 8:** Parameter estimates for the full sample model.

Factor code	Coefficient	S. E.	Wald	*p* value	95% CI		OR(EXP(B))
					lcl	ucl	
FAC_1	−0.037	0.109	0.116	0.733	−0.252	0.177	0.96
FAC_2	−0.055	0.105	0.274	0.601	−0.261	0.151	0.95
FAC_3	−0.184	0.104	3.1	0.078	−0.388	0.021	0.83
FAC_4	0.225	0.12	3.501	0.061	−0.011	0.461	1.25
FAC_5	0.152	0.11	1.922	0.166	−0.063	0.367	1.16
FAC_6	−0.191	0.104	3.377	0.066	−0.395	0.013	0.83
FAC_7	−0.072	0.108	0.448	0.503	−0.283	0.139	0.93
FAC_8	−0.382	0.113	11.371	0.001	−0.604	−0.16	0.68
FAC_9	−0.131	0.105	1.562	0.211	−0.336	0.074	0.88
FAC_10	0.069	0.111	0.387	0.534	−0.148	0.286	1.07
Gender = 0	1.006	0.227	19.588	0	0.561	1.452	2.73
Gender = 1	0a						
Age = 1	0.405	1.146	0.125	0.724	−1.841	2.651	1.5
Age = 2	1.222	0.656	3.474	0.062	−0.063	2.506	3.39
Age = 3	1.073	0.661	2.635	0.105	−0.223	2.368	2.92
Age = 4	0.569	0.646	0.776	0.378	−0.697	1.835	1.77
Age = 5	0.669	0.582	1.321	0.25	−0.472	1.809	1.95
Age = 6	0a						
AFI = I	−0.027	0.333	0.007	0.935	−0.679	0.625	0.97
AFI = II	0.373	0.287	1.692	0.193	−0.189	0.935	1.45
AFI = III	0a						
HR = 0	0.335	0.287	1.358	0.244	−0.228	0.898	1.4
HR = 1	0a						
EA = 1	−1.146	2.078	0.304	0.581	−5.218	2.927	0.32
EA = 2	−0.949	0.604	2.465	0.116	−2.133	0.236	0.39
EA = 3	−1.132	0.508	4.955	0.026	−2.128	−0.135	0.32
EA = 4	−0.566	0.443	1.627	0.202	−1.435	0.303	0.57
EA = 5	−0.57	0.348	2.691	0.101	−1.252	0.111	0.57
EA = 6	0a						
PC = 0	−0.357	0.271	1.736	0.188	−0.889	0.174	0.7
PC = 1	0a						
Chronic = 0	0.125	0.226	0.305	0.581	−0.318	0.568	1.13
Chronic = 1	0a						

Where AFI represents annual household income, which is an ordinal categorical variable, less than 30,000 yen = 1, 30–99,000 yen = 2, 100–149,000 yen = 3, 15–19,000 yen = 4, 20–39,000 yen = 5, 400,000 yen and above = 6. HR represents household registration, which is a unordered categorical variable, local = 1, Non-local = 0. EA represents levels of education, which is an ordinal categorical variable, primary school and below = 1, junior secondary school = 2, senior secondary school/vocational secondary school = 3, college/higher vocational colleges = 4, Bachelor’s degree = 5, postgraduate (master’s degree and above) = 6. PC represents private car ownership, which is a unordered categorical variable, own a car or more = 1, no car = 0.

Among four objective food environment factors, the neighborhood-level Retail Food Environment Index (FAC_3) exhibited negative associations with healthy weight outcomes (β = −0.184, SE = 0.104, *p* < 0.1, OR = 0.83), significant only at the 500-meter scale. A 1-unit increase in this index reduced healthy weight likelihood by 17%. This counterintuitive finding challenges existing literature that categorizes fresh food markets as health-promoting facilities (44, 45). Food Availability and Diversity (FAC_4) demonstrated positive associations (β = 0.225, SE = 0.12, *p* < 0.1, OR = 1.25) at the 500-1000m scale, suggesting moderate-scale food resource richness may enhance dietary choices. While perceived food availability and accessibility (FAC_8) significantly influenced weight outcomes (β = −0.382, SE = 0.113, *p* < 0.001, OR = 0.68), with each unit increase reducing healthy weight likelihood by 32%.

Unhealthy Dietary Behaviors (FAC_6) increased obesity risk (= − 0.191, SE = 0.104, *p* < 0.1, OR = 0.83), where each unit elevation decreased healthy weight probability by 17%. Notably, Healthy Food Intake (FAC_9) showed no statistical significance (*p* = 0.211), potentially reflecting nutritional inequality in community food environments that negates individual healthy consumption efforts.

In addition to this, there are other factors that can have an impact on the weight of the population. Gender and levels of education significantly moderated weight outcomes: Females showed 2.81-fold higher likelihood of maintaining healthy weight than males (*p* < 0.001), potentially reflecting gendered health behavior patterns (46). Bachelor’s/high-school educated groups exhibited lower weight health than postgraduates (*OR* = 0.31/0.54, *p* < 0.01), suggesting positive educational gradients in weight management. Non-significant factors included age, income, vehicle ownership, chronic conditions, and substance use.

#### 3.2.3 Obesogenic neighborhood typology

Based on regression outcomes from Section 3.2 (Table 8), we analyzed 164 weight-abnormal cases (overweight samples: *N* = 131, 79.8%; obesity samples: *N* = 33, 20.2%) from the full sample (*N* = 405). Key obesogenic factors (FAC_3, FAC_6, FAC_8) were extracted for cluster analysis, and the elbow method identified three clusters as optimal, corresponding to distinct obesogenic neighborhood types (Table 9). The results reveal three primary obesogenic community types within the survey sample: Type I-Objective deprived (*N* = 10, 6.0%), Type II-Objective overloaded (*N* = 37, 22.5%), and Type III-Objective overloaded-Dietary behavior integrated (*N* = 117, 71.3%).

**Table 9:** K-means clustering results.

Clustering results			
	Cluster I	Cluster II	Cluster III
500-meter modified retail food environment index	0.3165	0.8075	0.7776
Unhealthy eating behavior	0.6311	0.4577	0.8297
Perceived food availability and accessibility	0.5151	0.5195	0.5004
Number of cases	10	37	117
Total	164		
Type	Objective deprived	Objective overloaded	Objective overloaded-dietary behavior integrated

Type I communities (*N* = 10, 6.0%), categorized as Objectively resource-deficient type, demonstrated the lowest sample size and objective food environment index (FAC_3 = 0.3165) while maintaining normative levels in perceived healthy food accessibility (FAC_8 = 0.5151) and dietary behaviors (FAC_6 = 0.6311). Predominantly located in suburban areas, these communities exhibited underdeveloped urban infrastructure compared to central districts, coupled with significantly younger demographics (Mean Age Group = 4.04). The observed subject-object environmental cognition discrepancy may stem from rapid lifestyle transformations among Chinese youth, characterized by diversified food acquisition methods and emerging digital food environments (DFEs). Notably, food delivery services have effectively decoupled dietary accessibility from physical spatial constraints (47).

Type II communities (*N* = 37, 22.5%), identified as objectively resource-overload type, presented the highest objective retail food environment index (FAC_3 = 0.8075) with minimal unhealthy dietary behaviors (FAC6 = 0.4577), yet paradoxically exhibited elevated obesity risk (standardized OR = 1.38). This cohort demonstrates that prolonged exposure to food-dense environments elevates obesity susceptibility despite self-reported healthy dietary practices. Socioeconomically disadvantaged populations within these communities (Mean AHI Type = 1.59) potentially face dual challenges: cognitive biases in nutritional assessment and economic constraints limiting dietary diversity, typically manifesting as carbohydrate-dominated nutritional patterns that paradoxically exacerbate metabolic risks.

Type III communities (*N* = 117, 71.3%), classified as behavior-driven integrated type, constituted the predominant urban distribution. These communities exhibited elevated objective food environment indices (FAC_3 = 0.7776), maximal unhealthy dietary behaviors (FAC_6 = 0.8297), and intermediate perceived food accessibility (FAC_8 = 0.5004). The identified obesogenic pathway aligns with established food environment models, demonstrating synergistic effects of food swamp (excessive unhealthy food outlets) and food desert (limited healthy food access) configurations (48). Chronic exposure to this dual environmental stressor – nutritional scarcity perception amidst hyper-available obesogenic foodscapes – likely drives sustained unhealthy dietary patterns through environmental-behavioral interactions.

## 4 Discussion

This study investigates the impacts of objective food environments, perceived food accessibility, and dietary behaviors on residents’ weight outcomes at the community scale in Tianjin, China, while establishing an obesogenic community typology based on localized determinants. Results reveal significant associations between food environment characteristics, dietary patterns, and obesity risks. Increased proportions of fresh vegetable outlets, heightened perceived accessibility, and frequent unhealthy dietary behaviors were associated with elevated obesity risks, whereas greater food environment diversity correlated with reduced risks. Spatial analysis demonstrated scale-dependent heterogeneity in environmental effects. Using k-means clustering, three obesogenic community types were identified: the majority (71.34%) exhibited high objective environmental risks combined with prevalent unhealthy eating behaviors, reflecting a distinctive obesogenic landscape in middle-income urban settings. These findings highlight the necessity for policymakers and urban planners to prioritize context-specific strategies that integrate food environment optimization with behavioral interventions in health-conscious urban governance.

Our study reveals a paradoxical association between excessive community provision of fresh produce and increased obesity risk, which invites a reconsideration of the conventional health benefits attributed to the Retail Food Environment Index (RFEI) (49, 50) across both objective and perceived dimensions. Specifically, elevated modified Retail Food Environment Index (mRFEI) values within 500-meter buffers showed positive associations with obesity (*β* = −0.184, *p* = 0.078, OR = 0.83), while heightened perceived food accessibility similarly correlated with elevated obesity risk (β = −0.191, *p* = 0.066, OR = 0.83). Previous studies using either RFEI metrics or perceptual evaluations generally associate higher densities of fresh fruit and vegetable outlets with lower obesity risks (44, 45, 50–52) However, 90% of these studies were conducted in high-income countries such as those in North America and Europe, with no universally applicable standards due to contextual complexities (17). Both mRFEI and perceptual measures in our study indicate that fresh produce markets—retail food facilities conventionally deemed healthy—may unexpectedly elevate obesity risks. Parallel concerns emerge from a Guatemalan study revealing 42% misclassification errors in traditional RFEI’s “healthy” food outlet categorization (49). Research in Hong Kong also demonstrates significantly higher densities of both healthy and unhealthy food outlets compared to findings in the United States, United Kingdom, and Canada (53).

These disparities likely stem from China’s unique built environment, dietary habits, and food facility characteristics. Regarding the built environment, Chinese urban areas exhibit high-density development patterns that enhance accessibility to diverse food facilities. Government-led infrastructure initiatives, such as ubiquitous wet markets (54), render “food deserts” virtually nonexistent. Paradoxically, communities with high Retail Food Environment Index values often feature monotonous food supply options, potentially increasing obesity risks by limiting dietary diversity. In terms of dietary behavior, fresh produce markets—perceived as scarce “healthy facilities” in Global North studies (8, 55, 56)—are deeply embedded in Chinese daily life. Higher perceived accessibility to these facilities may amplify total caloric intake (regardless of food healthiness) through habitual purchasing patterns (57). Furthermore, Chinese fresh food retail outlets frequently sell both healthy items (e.g., vegetables and fruits) and energy-dense snacks (e.g., fried foods), complicating their health impacts through heterogeneous product offerings.

Another key finding reveals scale-dependent effects of neighborhood food environments on residents’ weight outcomes. Our analysis demonstrates that the obesogenic effects of the Retail Food Environment Index (RFEI) become insignificant at larger spatial scales (1000m radius), with obesity risk showing significant association only with facilities within the immediate living area (500m radius). Notably, improved food accessibility within the 500–1000m range significantly enhanced weight-related health outcomes. This spatial gradient aligns with existing evidence (50) documented similar scale dependence in Edmonton, Canada, where food environment impacts emerged at 800m but dissipated at 1600m. However, these spatial patterns exhibit geographic variability: Bodor et al. (44) identified significant obesogenic effects up to 2000m in New Orleans, while Acciai et al. (58) observed beneficial BMI associations with small grocery stores within 0.4km in New Jersey’s low-income communities. The 500-1000m health effect window in our study likely reflects China’s distinctive urban morphology characterized by gated residential communities. Typical Chinese neighborhood units (300-500m radius) concentrate daily amenities through planned development, creating concentrated foodscape exposures. This spatial configuration intensifies food environment impacts at intermediate scales, as residents’ routine activities remain anchored to these planned service clusters. The observed effects may stem from the compound interaction between objective deprivation (limited healthy options in immediate vicinity) and behavior-driven integrated factors (travel patterns constrained by community design).

Through K-means clustering analysis, we identified three distinct obesogenic community typologies in Tianjin. The most prevalent type, Type III-Behaviorally Dominant Composite Obesogenic, is characterized by abundant objective food environments yet persistent unhealthy dietary behaviors among residents. Type I communities, conversely, exhibit limited objective food infrastructure but still face obesity risks, often located in suburban areas with younger populations. Here, digital food environments (e.g., food delivery platforms) compensate for physical food access deficiencies (47). Meanwhile, Type II communities reveal a critical cognitive dissonance: residents self-report minimal unhealthy dietary behaviors despite elevated obesity risks, reflecting widespread misconceptions about healthy eating. In China, many individuals—particularly older adults and lower-income groups—equate high carbohydrate intake (e.g., rice, noodles) with nutritional adequacy, overlooking balanced protein, dietary fats, and fiber consumption (59). Compared to these studies, the addition of food environment indicators based on residents’ subjective perceptions in this study helps to bridge this gap.

Our findings reaffirm the established pathway linking unhealthy food environments and dietary behaviors to obesity in China, while also exposing a health perception gap between objective and perceived environments. This misalignment mirrors recent studies (60, 61), such as Philadelphia-based research demonstrating that perceived food environments better capture local fresh produce quality and affordability than objective metrics (62)—insights unattainable through purely environmental audits. Current obesogenic interventions, particularly those targeting nutritional inequality in developing countries, disproportionately emphasize improving healthy food accessibility or walkability. These results necessitate a paradigm shift for policymakers and planners: optimizing objective environments alone proves insufficient. We advocate for contextualized interventions that address both foodscape realities and residents’ health literacy, including:

- Redefining “healthy” food facilities through community-engaged assessments.
- Developing typology-specific strategies (e.g., digital food environment regulation for Type I; nutrition education for Type II).
- Integrating multi-scalar planning frameworks based on the link between physical infrastructure and behavioral factors.

This study has some limitations, which include the cross-sectional design’s inability to resolve endogeneity and omitted variables. First, the cross-sectional design of the questionnaire data limits causal inference due to potential reverse causality, which requires longitudinal or experimental designs to resolve. Second, while obesity involves multifactorial determinants, this analysis may omit other dimensions: (1) Built environment factors: open spaces, sports facilities, and street walkability. (2) Individual-level confounders: perceived environmental stress, regional dietary preferences, and household food cultures. (3) Geographical spillovers: workplace/school food environments.

Despite these constraints, the study illuminates middle-income countries’ unique obesogenic foodscapes, offering critical insights for health-promoting urban governance. Future research should adopt longitudinal designs and expand geographical samples to disentangle the complex interplay between food environments, cultural norms, and dietary transitions in Global South countries.

## Data Availability

The raw data supporting the conclusions of this article will be made available by the authors, without undue reservation.

## Footnotes

^1^ https://www.resdc.cn/
