# Supplementary material for: Unveiling the obesogenic neighborhood food environment factors and typologies in Tianjin, China: an integrative analysis of perceived and objective measures
Source: Front Public Health. 2025 Nov 21;13:1665021. doi: 10.3389/fpubh.2025.1665021 (PMC12678105; doi:10.3389/fpubh.2025.1665021)
Supplement: Supplementary file 1 [file Table_1.docx]

Supplementary Material

# Informed Consent Form for Questionnaire Survey

Dear Residents and Participants,

Thank you for your participation. This document, issued by the Research Section of Environment Design at Dalian University of Technology, outlines the purpose, content, privacy protection measures, and relevant details of the "Community Food Environment and Healthy Dietary Behaviors Survey" conducted by the RSED Institute. Please review this information carefully to ensure your fully informed and voluntary participation.

## Research Purpose and Scope

This survey aims to investigate the environmental quality of healthy food retail facilities in your community and individual health-related dietary behaviors. Collected data will be analyzed to formulate evidence-based intervention measures. The questionnaire may involve personal information requiring confidentiality. As a representative of public opinion, your input holds critical guiding significance. We strictly adhere to national confidentiality regulations for social surveys and guarantee that all personal information will remain inaccessible to any parties beyond the research team.

## Privacy Protection Protocol

All personal identifiers will be anonymized throughout the research process (including data collection, data cleansing, and data analysis phases). The anonymized dataset will be utilized solely for academic research purposes, with robust measures implemented to prevent information disclosure.

## Participant Rights

You retain the right to:

- Decline answering any questions deemed sensitive
- Terminate participation at any stage
- Request access to your personal information collected herein
- Timeline and Contact Information
  This survey will be conducted over an 8-week period. For inquiries or assistance, please contact:

Ms.Yue Sun | +86-158-XXXX-1834

Research Section of Environment Design

School of Architecture and Fine Art

Dalian University of Technology

January 2, 2023

# Questionnaire

## Basic Information Survey

1. What is your gender?

A. Male B. Female

2. Which of the following age ranges are you in?

A. Below 18 years old B. 18~29 years old C. 30~39 years old D. 40~49 years old

E. 50~59 years old F. 60 years old and above

3. Location of the neighborhood you live in: _________

4. Number of people in your household: _________

5. Your annual household income is about

A. less than 30,000 yuan B. 30~99,000 yuan C. 10~149,000 yuan D. 15~199,000 yuan

E. 20~399,000 yuan F. More than 400,000 yuan

6. Are you a local?

A. Yes B. No

7. Your level of education is

A. Elementary school or below B. Middle school C. High school or junior college D. College E. Bachelor's degree F. Graduate school or above

8. Your employment status is

A. Employed (including school students, temporary employment) B. Unemployed, unemployable, laid off or retired

## Community Food Environment Perception Survey

9*. Tianjin is a famous (Please choose “Civilized city” for this question regardless of what you think the result is)

A. Industrial city

B. Port city

C. Civilized city

D. A new first-tier city

*Note:**  *refers to an attention span test question used to screen the results of a questionnaire on an online platform.*

10. Do you agree with these statements about the food retail facilities around your home? (Scale of 1 to 7, with 1 indicating totally disagree and 7 indicating totally agree.)

|  | 1 | 2 | 3 | 4 | 5 | 6 | 7 |
| --- | --- | --- | --- | --- | --- | --- | --- |
| Nutritious food can be easily purchased in this neighborhood, e.g. a full range of foods such as staple foods, main dishes and side dishes. | ○ | ○ | ○ | ○ | ○ | ○ | ○ |
| It is easy to buy daily food on foot, and there is good transport to get to the food facilities, so there are no inconveniences in daily shopping | ○ | ○ | ○ | ○ | ○ | ○ | ○ |
| Nutritionally balanced food is available in the neighborhood at more affordable prices | ○ | ○ | ○ | ○ | ○ | ○ | ○ |
| The opening hours and service of facilities such as supermarkets or grocery shops are satisfactory when buying necessary ingredients/food in the neighborhood | ○ | ○ | ○ | ○ | ○ | ○ | ○ |
| The environmental quality of facilities such as supermarkets or grocery shops is satisfactory when buying necessary ingredients/food in this neighborhood | ○ | ○ | ○ | ○ | ○ | ○ | ○ |
| Satisfactory service quality of facilities such as supermarkets or grocery shops when buying necessary ingredients/food in this neighborhood | ○ | ○ | ○ | ○ | ○ | ○ | ○ |
| The quality and appearance of ingredients/food bought in this neighborhood are satisfactory. | ○ | ○ | ○ | ○ | ○ | ○ | ○ |
| I feel confident that there are trustworthy merchants and producers in this neighborhood in terms of food safety | ○ | ○ | ○ | ○ | ○ | ○ | ○ |

11. Do you agree with the following statements about the food service facilities around your home? (Scale of 1 to 7, with 1 indicating totally disagree and 7 indicating totally agree.)

|  | 1 | 2 | 3 | 4 | 5 | 6 | 7 |
| --- | --- | --- | --- | --- | --- | --- | --- |
| There are a lot of restaurants around where I live that are easy to find that offer a full range of foods that are nutritious e.g. starters, mains and side dishes; | ○ | ○ | ○ | ○ | ○ | ○ | ○ |
| It is easy to walk to nearby restaurants and there is good transport to restaurants, so there are no inconveniences to daily meals R3-I can buy well-balanced food at a relatively affordable price at nearby restaurants | ○ | ○ | ○ | ○ | ○ | ○ | ○ |
| I can buy well-balanced food at a reasonable price at nearby restaurants. | ○ | ○ | ○ | ○ | ○ | ○ | ○ |
| Satisfactory opening hours and service when I want to eat in a restaurant. | ○ | ○ | ○ | ○ | ○ | ○ | ○ |
| The quality of the environment in the restaurant is satisfactory when I want to eat in the restaurant | ○ | ○ | ○ | ○ | ○ | ○ | ○ |
| The level of service quality in the restaurant is satisfactory when I dine in the restaurant | ○ | ○ | ○ | ○ | ○ | ○ | ○ |
| The quality and taste of ingredients/dishes served in restaurants in this neighborhood is satisfactory | ○ | ○ | ○ | ○ | ○ | ○ | ○ |
| Feeling confident about the food safety in the neighborhood, with more established businesses and producers that I can trust. | ○ | ○ | ○ | ○ | ○ | ○ | ○ |

## Dietary Behavior Survey

12. How rich is your average weekly food intake? (“Abundance” refers to the variety of food types, e.g., consuming enough meat, eggs, milk, seafood, staple foods such as grains, good fats, vitamins and other multi-category foods every day is called abundance, while consuming only one or two types of food such as carbohydrates or fats every day is called mono).

Very mono ○1 ○2. ○3. ○4. ○5. Very abundant

13. The frequency of your daily intake of healthy foods such as fresh fruits and vegetables is: _________ times/week, and the amount of intake: ___ grams/time.

Your weekly frequency of consuming sweets, sugary drinks, pickled and fried foods and fast food, street food is: _______ times/week, intake: ___ grams/time.

14. How do you most often buy to obtain food?

A. Offline direct purchase B. Online reservation/purchase

15. Do you most often buy ingredients to cook at home or eat out (take out food from restaurants)?

A. Cooking at home B. Eating out (or taking out food from restaurants)

16. Where do you usually go to buy food/ingredients for your daily needs? [Multiple choice]

A. General supermarkets

B. Vegetable market/farmer's market

C. Community supermarkets/grocery stores (e.g. community vegetable stores, mini-markets, etc.)

D. Convenience stores (e.g., Rosen, Express, 711, etc.)

E. Fresh food specialty stores

17. Where do you go most often to solve your dining problem [Multiple choice]

A. Chinese food Restaurant (e.g. Chinese restaurants, hot pots, etc.)

B. Western food Restaurant (e.g. Mexican restaurant, Italian restaurant, etc.)

C. Chinese fast food restaurant (such as Sha Xian snacks, cassoulet, chicken pot, etc.)

D. Western-style fast food restaurants (such as McDonald's, Wallace, Burger King, etc.)

E. Take-out Restaurants (e.g. Pancakes & Fruit, Meat Burrito, etc.)

F. Dessert Stores (e.g. bakeries, coffee, milk tea stores, etc.)

## Health Status Survey

18. Your height: _________ cm, your weight: ___Kg.

19. Do you have any of the following conditions?

|  | Yes | No |
| --- | --- | --- |
| Do you smoke | ○ | ○ |
| Do you drink alcohol | ○ | ○ |
| Do you own a car | ○ | ○ |
| Do you do housework | ○ | ○ |
| Do you suffer from chronic diseases (including respiratory diseases, cardiovascular diseases, immune diseases, metabolic diseases, digestive system diseases.) | ○ | ○ |

20. Your daily frequency of moderate to high intensity physical activity is _______ times/week; the average duration of each exercise is ____min. (Note: Moderate-intensity physical activity such as jogging, walking up the stairs, biking uphill, skating, volleyball, hiking, etc.) If you do not exercise daily, please put 0 for both. High-intensity physical activity such as long-distance running, jumping rope, weight lifting, fencing, etc.) If you do not exercise on a daily basis, please fill in 0 for all of them.

21. What is your self-assessed health status (on a scale of 1 to 5. 1 means unhealthy, 2 means sometimes sub-healthy, 3 means average, 4 means healthy, 5 means very healthy)?

A. unhealthy B. sometimes sub-healthy C. average D. healthy E. very healthy
